# Insulin Signal Transduction Perturbations in Insulin Resistance

**DOI:** 10.3390/ijms22168590

**Published:** 2021-08-10

**Authors:** Mariyam Khalid, Juma Alkaabi, Moien A. B. Khan, Abdu Adem

**Affiliations:** 1 Departments of Pharmacology, College of Medicine and Health Sciences, United Arab Emirates University, Al Ain P.O. Box 17666, United Arab Emirates; 201690406@uaeu.ac.ae; 2 Departments of Internal Medicine, College of Medicine and Health Sciences, United Arab Emirates University, Al Ain P.O. Box 17666, United Arab Emirates; j.kaabi@uaeu.ac.ae; 3 Departments of Family Medicine, College of Medicine and Health Sciences, United Arab Emirates University, Al Ain P.O. Box 17666, United Arab Emirates; moien.khan@uaeu.ac.ae; 4 Department of Pharmacology, College of Medicine and Health Sciences, Khalifa University, Abu Dhabi P.O. Box 127788, United Arab Emirates

**Keywords:** insulin resistance, hyperglycemia, type 2 diabetes mellitus, insulin signaling, pancreatic beta cells

## Abstract

Type 2 diabetes mellitus is a widespread medical condition, characterized by high blood glucose and inadequate insulin action, which leads to insulin resistance. Insulin resistance in insulin-responsive tissues precedes the onset of pancreatic β-cell dysfunction. Multiple molecular and pathophysiological mechanisms are involved in insulin resistance. Insulin resistance is a consequence of a complex combination of metabolic disorders, lipotoxicity, glucotoxicity, and inflammation. There is ample evidence linking different mechanistic approaches as the cause of insulin resistance, but no central mechanism is yet described as an underlying reason behind this condition. This review combines and interlinks the defects in the insulin signal transduction pathway of the insulin resistance state with special emphasis on the AGE-RAGE-NF-κB axis. Here, we describe important factors that play a crucial role in the pathogenesis of insulin resistance to provide directionality for the events. The interplay of inflammation and oxidative stress that leads to β-cell decline through the IAPP-RAGE induced β-cell toxicity is also addressed. Overall, by generating a comprehensive overview of the plethora of mechanisms involved in insulin resistance, we focus on the establishment of unifying mechanisms to provide new insights for the future interventions of type 2 diabetes mellitus.

## 1. Introduction

Type 2 diabetes mellitus (T2DM) is a serious medical challenge of the 21st century. Persistently elevated glucose concentrations above the physiological range result in the manifestation of diabetes. The peptide hormone insulin released from pancreatic β-cells maintains normal blood glucose levels by regulating whole-body carbohydrate, lipid, and protein metabolism [1]. Insulin performs its function through signal transduction in insulin-responsive tissues, specifically the liver, skeletal muscles and adipose tissues as these tissues play a distinct role in the regulation of metabolic homeostasis [2,3].

Insulin resistance is the condition in which a cell, tissue, or body of an organism cannot adequately respond to normal levels of insulin. This in turn hinders insulin’s function of maintaining glucose and lipid homeostasis [4]. The inability of insulin to provide normoglycemia leads to compensatory hyperinsulinemia, which further enhances insulin secretion from β-cells. Insulin secretion for a prolonged period exhausts pancreatic β-cells and leads to their apoptosis [5,6]. Insulin resistance also promotes gluconeogenesis in the liver, disrupts glucose uptake in muscles, and induces lipolysis in adipose tissues. Insulin resistance may be developed through both genetic and acquired factors [7]. The common genetic defects include mutations and polymorphism of insulin receptors, glucose transporters, and signaling proteins involved in insulin signal transduction. The acquired causes of insulin resistance include obesity, physical inactivity, advanced glycation end products (AGE), excess free fatty acids (FFAs), psychological stress, smoking, alcohol intake, or certain medications [8,9,10]. All these factors are linked to constant low-grade inflammatory conditions [11,12]. The sustained elevation of interleukin-1 beta (IL-1β), interleukin-6 (IL-6), tumor necrosis factor-α (TNF-α), or FFAs impairs the balance of secretion between inflammatory and anti-inflammatory cytokines. This causes accumulation of unfolded protein, leading to activation of unfolded protein response (UPR), induction of endoplasmic reticulum stress (ER stress), and oxidative stress, which further impairs insulin sensitivity [13,14,15].

In this review, we mainly focus on the perturbations of downstream critical nodes of the insulin signaling mechanisms in an insulin-resistant state. Insulin resistance is a consequence of a complex combination of metabolic disorders, lipotoxicity, glucotoxicity, and inflammation. At the insulin receptor site, the insulin signal transduction includes various upstream and downstream modulator proteins. Alterations in the regulation of insulin signaling mechanisms are the final manifestation of insulin resistance [16,17,18]. Here, we attempt to unravel the detailed circuitry of the PI3K-Akt signaling network concerning pathological features elicited by its dysregulation to guide further mechanistic investigations. Special attention is given to mediators that link cellular mechanisms associated with the pathophysiology of insulin resistance. This review aims to provide a better understanding and insight for the future interventions of diabetes mellitus. Treatments should not only target the insulin signaling pathway perturbations but should also aim at the AGE-RAGE-NF-κB axis, lipotoxicity, inflammation, and oxidative stress. In this review, we hope that a more sophisticated understanding of the mechanistic link between insulin resistance and its associated key pathophysiological processes presents the molecular basis to facilitate the development of novel therapies for type 2 diabetes mellitus.

## 2. Insulin Receptor and Insulin Signaling Pathway

**Figure 1:**
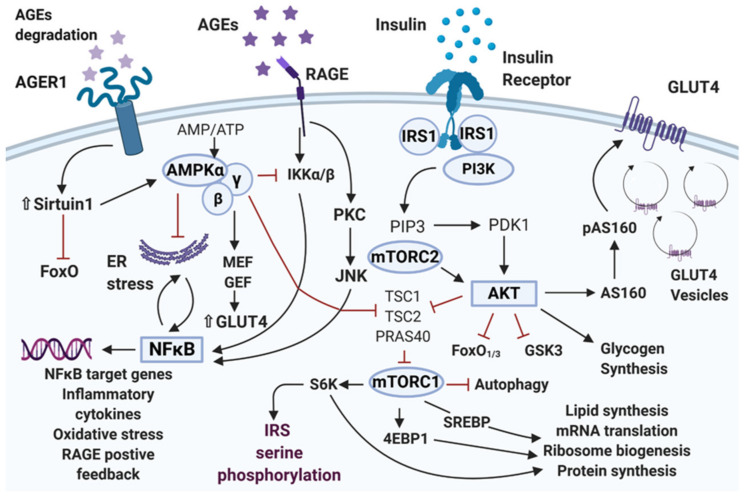
Various pathways involved in dysregulation of insulin signaling. Upon insulin binding, insulin receptor gets autophosphorylated. This recruits different substrate adaptors for the signal transduction. The tyrosine phosphorylated IRS1 recruits PI3K, which phosphorylates the serine/threonine residue of protein kinase B (Akt). Akt regulates the translocation of glucose transporter GLUT4 to the cell surface through phosphorylation of the GTPase-activating protein (AS160). Akt promotes glycogen synthesis through inhibition of GSK3 activity and induces protein synthesis via activation of mTOR and downstream elements. Akt phosphorylates and directly inhibits FoxO transcription factors, which inhibits autophagy. The hyperglycemia-induced production of AGE and binding with their receptor RAGE impairs insulin signal transduction by triggering a range of signaling pathways, including JNK, NF-κB, and activation of PKC. The sustained accumulation of AGE depletes the expression of anti-AGE cell surface receptor AGER1, which is responsible for inhibiting the deleterious effects of AGE by competitively interfering with its binding to RAGE. AGER1 along with Sirtuin1 promotes AMPK phosphorylation and activation, which induces GLUT4 gene expression through activation of MEF, GEF transcription factors. The AGE-RAGE induced activation of PKC, NF-κB mediated inflammation, and oxidative stress promotes the serine phosphorylation of IRS, inhibits its action, and induces insulin resistance. AGE: Advanced glycation end products; AGER1: AGE receptor 1 encoded by DDOST gene; AMPK: AMP-activated protein kinase; AS160: Akt substrate of 160 kDa; FoxO: Forkhead family of transcription factors; GLUT4: Glucose transporter protein 4; GEF: GLUT4 enhancer factor; GSK3: Glycogen synthase kinase 3; IKK: IκB kinase; IRS: Insulin receptor substrate1; MEF: Myocyte enhancer factor; mTOR: Mammalian target of rapamycin NF-κB: Nuclear factor κB; PDK1: Phosphoinositide-dependent kinase 1; PI3K: Phosphoinositide 3-kinase; PIP3: Phosphatidylinositol (3,4,5)-trisphosphate; PKC: Protein kinase C; RAGE: Receptor for advanced glycosylation end products; S6K: Ribosomal protein S6 kinase; SREBP: Sterol regulatory element-binding proteins; JNK: c-Jun N-terminal kinase.

Insulin receptor (IR) is a tyrosine kinase receptor that undergoes autophosphorylation upon insulin binding. Structurally, IR is a transmembrane heterotetrameric glycoprotein that has two α and two β subunits linked with a disulfide bond [19]. The IR is virtually expressed on the surface of all tissues. Major targets are insulin-responsive organs such as the liver, skeletal muscle, and adipose tissue [20]. The IR has an extracellular portion that is associated with insulin binding and an intracellular portion that is associated with its tyrosine kinase activity. As the insulin binds to the extracellular portion of the α subunit, oligomerization of the receptor and autophosphorylation of its tyrosine residues amplifies the kinase activity of the IR [21,22]. Along with the phosphorylation of IR, insulin receptor substrate (IRS), Casitas B-lineage lymphoma (Cbl), or Cbl associated proteins, which are the scaffolding proteins, also get phosphorylated and bind to intracellular receptor sites [23]. The IRS is the connecting molecule that, after phosphorylation, recruits signaling molecules to generate the insulin signal response and promote the activation of different downstream signaling pathways as the phosphoinositide-3-kinase (PI3K) or the CAP/Cbl/TC10 pathway. PI3K is a class 1 phosphoinositide 3-kinase and once activated causes the generation of phosphatidylinositol (3,4,5)-trisphosphate (PIP3), which has multiple effects on insulin-dependent glucose metabolism [17] (Carnagarin et al., 2015). PIP3 docks the pleckstrin homology (PH) domain containing protein kinases, which, in turn, activates phosphoinositide-dependent kinase 1 (PDK1) and protein kinase B or Akt kinase [17,24]. Upon activation, Akt migrates to the plasma membrane and phosphorylates Akt substrate of 160 kDa (AS160) [25,26]. As a consequence, glucose transporter protein (GLUT) is trafficked from intracellular storage vesicles to the plasma membrane [27] (Figure 1). Insulin-mediated glucose uptake and storage under anabolic conditions in insulin-sensitive tissues are facilitated by the recruitment of GLUT to the cell surface, which is governed by distinct signaling pathways and is crucial for insulin sensitivity [25,28].

## 3. Mechanisms Involved in Insulin Resistance

### 3.1. β-Cell Function and Mass

Insulin secretion and insulin sensitivity are regulated by pancreatic β-cells in a very definite manner to maintain homeostatic concentrations of plasma glucose in healthy individuals. During normal physiological conditions, there is a positive feedback loop between the β-cells and insulin-sensitive tissues with enhanced insulin supply by β-cells in response to demand by the liver, skeletal muscle, and adipose tissue [5,29]. The magnitude of insulin response from β-cells depends on the sensitivity of insulin-responsive tissues [30,31]. The intimate, highly complex link between insulin resistance and β-cell dysfunction has important roles in triggering the pathogenesis of type 2 diabetes mellitus. The exhaustion of β-cells to maintain euglycemia and compensate for insulin demand in the insulin-resistant state is critical to the pathogenesis of the condition [29].

Both in rodents and humans, FFAs are crucial for glucose-stimulated insulin secretion (GSIS) from pancreatic β-cells through to the activation of G-protein coupled receptors specifically GPR40 [32]. During the insulin-resistant phase, which is characterized by lipotoxicity and elevated FFAs, overstimulation of glucose-mediated insulin secretion in β-cells results in increased β-cell signaling and oxidative stress, which leads to metabolic exhaustion of beta cells [33,34]. Glucose along with elevated FFAs leads to an increase in β-cell mass by increasing IRS2 expression of β-cells, which is associated with neogenesis, proliferation, and survival of pancreatic β-cells as a compensatory mechanism for insulin resistance. However, the resulting hyperinsulinemia leads to β-cell dysfunction [35]. The ratio of proinsulin and insulin split products relative to total immunoreactive insulin is increased in type 2 diabetes mellitus patients reflecting their β-cell dysfunction compared with healthy individuals [36]. The deposition of islet amyloid polypeptide (IAPP) or amylin in pancreatic islets is the other pathological mechanism involved in β-cell dysfunction. IAPP deposition causes a progressive decline in pancreatic islet cell mass, predominantly β-cells. Recently it was identified that the increase in expression of the receptor for advanced glycation end products (RAGE) contributes to IAPP induced β-cell proteotoxicity, as RAGE by binding to IAPP toxic intermediates triggers oxidative stress, inflammation, and apoptosis that are known to be upregulated in IAPP induced β-cell pathogenesis [37]. The accumulation and binding of ligands, i.e., AGE and IAPP toxic intermediates, upregulate RAGE expression and transduce RAGE-mediated intracellular signaling, leading to islet inflammation and β-cell apoptosis [33,37,38].

Increased cellular glucose metabolism, accumulation of saturated long chain fatty acid signaling, defective proinsulin processing, abnormal insulin secretion, the decline in β-cell mass, and islet amyloid deposition are some of the key factors in an insulin-resistant state that contribute to β-cell demise and lead to disease progression [39].

### 3.2. Insulin Receptor Substrate

The IRS is a critical mediator of insulin action and is a major site for both positive and negative regulation of the insulin signaling pathway. The IRS family has two major proteins IRS1 and IRS2, which function as docking proteins between the insulin receptor and intracellular signaling cascade [40,41]. IRS1 and IRS2 are significantly divergent in their abundance and tissue-specific function. Significantly lower expression of IRS1 has been observed in morbidly obese and insulin-resistant subjects [42]. IRS1-dependent insulin signal transduction is predominant over IRS2 in skeletal muscle, which has a significant role to maintain whole-body metabolism [43]. Insulin-mediated IRS tyrosine phosphorylation is the key intermediate in insulin signal transduction and serves as the major target for insulin resistance inducers [41]. Under normal physiological conditions, the insulin signaling also induces the IRS1 serine phosphorylation at specific sites as a negative feedback control mechanism through the activation of several kinases. Regulation of insulin/IGF-1 signaling by IRS involves phosphorylation at serine/threonine residues, dephosphorylation by the phosphatase, and degradation through the ubiquitin-proteasome [20,44].

The gene encoding IRS1 is highly polymorphic, and some notable single nucleotide polymorphisms (SNPs) are reported in insulin-resistant subjects [43,45]. These mutations reduce the extent of phosphorylation of IRS1 and the insulin stimulated PI3K activity, leading to impaired insulin action. In insulin resistance, IRS1 malfunctions more often than IRS2 [40,41]. IRS1 and IRS2 share a high degree of structural homology, but they differ in their activation sites and cellular localization [40]. IRS1 has an N-terminal PH domain, which is flanked by the phosphotyrosine binding (PTB) domain, responsible for its interaction with IR. The tail of IRS1 contains distinct tyrosine and serine phosphorylation sites, which serve as the docking locus for SH2 homology signal transducers [20,46]. The “diabetogenic” factors, specifically FFAs, inflammatory cytokines, reactive oxygen species, and hyperinsulinemia, strikingly increase various serine kinases, which include IκB kinase (IKK), c-Jun N-terminal kinase (JNK), specific isoforms of protein kinase C (PKC), double-stranded RNA-dependent protein kinase (PKR), and Rho-associated coiled-coil containing protein kinase (ROCK) [47]. These stress kinases not only hamper the IRS1 function but promote insulin resistance through upregulation of the expression of genes involved in the activation of inflammation and nuclear factor kappa B (NF-κB), thus further intensifying the problem. Phosphorylation of IRS1 at its serine residues, specifically on Ser**^307^**, Ser**^612^**, and Ser**^632^**, serves as a core element in the development of insulin resistance [41]. This phosphorylation cascade is a complex regulatory process that inhibits IRS1 tyrosine phosphorylation resulting in dissociation of the IR:IRS1 complex and promotes IRS1 de-localization and degradation [41,48]. In the light of above-mentioned data, the strategic intervention that can upregulate the expression of IRS1 and has functional significance can alleviate insulin resistance-induced skeletal muscle pathological damage. Moreover, detection of IRS1 novel variants that substantially contribute to the development of insulin resistance needs to be assessed.

### 3.3. Phosphatidylinositol 3-Kinase

Following the tyrosine-phosphorylation of IRS, Phosphatidylinositol 3-kinase (PI3K) is an important effector molecule. PI3K is a heterodimer having serine kinase activity, which enables it to interact with other signaling proteins [49]. PI3K is a key regulatory node in translating extracellular signals activated by insulin and growth factors into intracellular actions [50]. The class 1A PI3K comprises two subunits, 85-kDa regulatory subunit and a 110-kDa catalytic subunit. The regulatory subunit is responsible for the negative regulation of PI3K and has three isoforms: p85α, p85β, and p55γ [17]. The p85 subunit interacts with tyrosine-phosphorylated IRS through its two SH2 domains, and the p110 subunit hydrolyzes the membrane-bound phosphatidylinositol 4,5-bisphosphate (PIP2). The conversion of PIP2 to PIP3 is crucial for the recruitment of PDK1, which is responsible for phosphorylation of Akt that stimulates insulin signal transduction [51].

The balanced expression level of the p85 monomer and p85-p110 heterodimer is a critical feature of PI3K activation [52,53]. Insulin resistance is mediated when the expression of p85α monomer is enhanced as it competes with p85-p110 heterodimer and sequesters IRS1 and recruits inhibitory proteins responsible for PIP3 degradation. The p85α is encoded by PIK3R1. Mutations in PI3KR1 are associated with reduced PI3K signaling and an escalated risk of type 2 diabetes mellitus [54,55]. The combined outcome of enhanced p85 subunit expression and IRS serine phosphorylation is adequate to develop clinical insulin resistance [17,50,56].

Further investigation of the mechanisms that hamper binding of the p85-p110 heterodimer to the insulin receptor, block the activation of catalytic subunit p110, and cause mutations in the regulatory subunit of PI3K may serve as a guide for finding possible molecular targets for the prevention of impaired insulin signaling.

### 3.4. Protein Kinase B/Akt

The serine/threonine protein kinase Akt (also known as protein kinase B or PKB) is a member of the AGC protein kinases family. Akt consists of three homologous isoforms, i.e., Akt1, Akt2, and Akt3. The three isoforms share structural similarities but exhibit diverse target specific roles [57]. Among all the isoforms of Akt, Akt2 is the most important one in insulin-mediated glucose uptake and lipid metabolism. PDK1 initiates the Akt activation by phosphorylation of Akt at its threonine residue; subsequently, mTORC2 completes the Akt activation process by phosphorylation of Akt at its serine residue [25,58]. Phosphorylation at Thr**^308^** and Ser**^473^** is required for the maximal activation of Akt. Akt directly phosphorylates AS160, limiting the activity of the Rab-GTPase activating protein, leading to GLUT4 translocation and glucose uptake. Akt-mediated activation of mTORC1 through inhibition of the tumor suppressor complex (TSC1/TSC2) regulates several proteins involved in protein synthesis, including ribosomal protein p70, ribosomal S6 kinase 1 (S6K1) and the eukaryotic initiation factor 4E (eIF4E)-binding protein 1 (4E-BP1), by the acceleration of mRNA translation [17]. Akt exerts an inhibitory effect on glycogen synthase kinase 3 (GSK3) by phosphorylation resulting in glycogen synthase activation. Akt through mTORC1 activation suppresses lipolysis and promotes lipid biosynthesis by regulating the sterol regulatory element-binding protein (SREBP) substrate. Akt interacts and phosphorylates forkhead box transcription factor (FoxO) proteins, which are involved in the regulation of lipogenic and gluconeogenic gene expression, particularly FoxO1 and FoxO3. Akt attenuates the transcriptional activity of FoxO1 through its translocation from the nucleus, thus reducing glucose levels [59,60,61] (Figure 1).

Akt plays a crucial role in cell survival and growth through its action on insulin-stimulated glucose uptake, glycogen, and protein synthesis. The depletion of p-Akt expression is physiologically regulated for metabolic homeostasis either through the negative feedback by S6K1 or by the intracellular signaling molecules known as inositol phosphates [62,63]. The inositol phosphate, specifically inositol pyrophosphate IP7 generated by inositol-hexakisphosphate (IP6) kinase 1 (IP6K1), directly by its binding to the PH domain of Akt competes for PIP3 binding to Akt [64]. Studies have shown that the IP6K1-deleted mice phenotype shows enhanced Akt activity, sustained insulin sensitivity, and resistance to obesity as compared to its wild type [64,65]. Deregulated inositol metabolism leads to Akt inhibition and is associated with hyperglycemia and insulin resistance [66]. It is evident from both human and animal research that knockdown or depletion of either Akt1 or Akt2 isoform causes glucose intolerance, insulin resistance, and diabetes-like symptoms. Any defect in the PI3K/Akt/AS160 signaling will eventually reduce the glucose uptake in insulin-sensitive tissues and lead to insulin resistance [21,67].

Since the discovery of Akt in the early 1990s, a remarkable understanding of Akt’s role in the regulation, function, and manipulation of insulin signal transduction has been made. The cross-talk of Akt with other major cellular signaling networks has indicated Akt as an attractive candidate for further mechanistic exploration. In this regard, regulatory phosphorylation events on Akt need to be explored as most studies have been centered on two major regulatory sites Thr**^308^** and Ser**^473^** on Akt.

### 3.5. GLUT4

Glucose transporter proteins (GLUTs) are fundamental membrane proteins that are responsible for glucose transport across the plasma membrane into cells. There are 13 glucose transporter proteins expressed in distinct tissues having different kinetics and respective substrate specificities. GLUT4 is the predominant insulin-responsive glucose transporter responsible for the regulation of glucose disposal to maintain whole-body glucose homeostasis [68,69]. Upon insulin signal transduction, GLUT4 translocates from intracellular storage vesicles to the cell membrane to enhance glucose uptake. The expression levels of GLUT4 mRNA and protein affect insulin sensitivity and can modify whole-body metabolism [70]. It has been shown that the condition of insulin deficiency or resistance can result in depletion of GLUT4 expression in skeletal muscle due to transcriptional repression as in T1DM and T2DM. NF-κB, characterized as the mediator of TNF-α, is an important inhibitor of GLUT4 transcription. Exercise and muscle differentiation are the potent physiological stimulus for upregulation of GLUT4 expression and translocation [71,72,73].

Different research studies conducted both in tissue culture cell lines and in transgenic mice have demonstrated that the regulation of GLUT4 gene expression is under the dynamic control of specific transcription factors, the GLUT4 enhancer factor (GEF), and myocyte enhancer factor 2 (MEF2) [74]. The binding of heterodimer MEF2A-MEF2D to the consensus sequence in the GLUT4 promoter region upregulates GLUT4 expression and any deletion or truncation of that specific consensus sequence leads to repression in GLUT4 mRNA expression [71,75]. The transcriptional activation of MEF2 is regulated by diverse mechanisms, such as calcium levels in the muscle, dephosphorylation by calcineurin, and AMPK activation, which increases PPARβ via MEF2A [76,77].

As GLUT4 expression is much decreased in insulin-resistant state and T2DM, potent stimulators of GEF and MEF2 can be used as an alternative treatment to enhance GLUT4 expression for improved insulin sensitivity as experimentally demonstrated in some research studies through the use of GLUT4 overexpression in murine skeletal muscle [70,77,78]. Studies have shown that moderate overexpression of GLUT4 in both in vitro and in vivo models positively manipulates glucose uptake and alleviates lipotoxicity [79,80].

Despite intensive efforts to understand the GLUT4 pathway, certain gaps in the regulation of GLUT4 trafficking and signaling components involved in the regulation of GLUT4 translocation remain elusive. Future investigation to better understand molecular details of GLUT4 regulation may lead closer to the advent of selective pharmacological intervention.

### 3.6. Mammalian Target of Rapamycin/mTOR

The mammalian target of rapamycin (mTOR) regulates cellular growth and metabolism through its serine/threonine kinase activity. mTOR forms two functionally distinct complexes: mTOR complex 1 (mTORC1) and mTOR complex 2 (mTORC2). Both these multi-protein complexes are activated by growth factors and insulin [81,82].

mTORC1 consists of five components. Insulin-IRS mediated activation of Akt results in indirect activation of mTORC1, which, in turn, phosphorylates two key components; the 4E-BP1 and the S6K1. Upon phosphorylation, 4EBP1 dissociation from eIF4E allows eIF4E to promote 5′ cap-dependent mRNA translation leading to protein synthesis. Activation of S6K1 by mTORC1 not only leads to an increase in mRNA biogenesis and translation initiation but is also involved in negative feedback regulation of insulin signaling through serine/threonine phosphorylation of IRS, which ultimately leads to IRS depletion [83,84,85] (Figure 1). Chronic activation of mTORC1, as in the case of overnutrition or obesity, leads to insulin resistance through a negative feedback loop through IRS degradation. One study conducted in mice deficient in S6K1 demonstrated that mice were protected against insulin resistance under high-fat diet (HFD) conditions, which was apparent in the loss of negative regulation from S6K1 through its serine phosphorylation of IRS1 [86,87]. In line with the negative feedback role of mTORC1/S6K1, it has a crucial role in the growth and survival of β-cell and insulin secretion. β-cell mass is reduced in chronic administration of rapamycin (inhibitor of mTOR) in rats, and S6K1 knockout mice tend to have small-sized pancreatic β cells [84].

The mTORC2 complex consists of seven protein components, most of which are similar to mTORC1. In comparison to mTORC1, little is known about the mTORC2 pathway. mTORC2 plays a positive role in cell survival and cytoskeleton organization. As mTORC2 activation is dependent on the insulin-PI3K transduction pathway, that is why it is affected by the mTORC1/S6K1 negative feedback loop through phosphorylation of mammalian SAPK interacting protein 1 (mSin1), a key component of mTORC2 [88]. Partially active Akt promotes the activation of mTORC2, which, in turn, phosphorylates several kinases, including Akt and protein kinase C α (PKCα). mTORC2 regulates glucose homeostasis through Akt, which is involved in GLUT4 translocation and deactivation of GSK-3, thus decreasing the rate of phosphorylation of glycogen synthase. This leads to enhanced glycogen accumulation, particularly in the liver and muscles. Akt also controls glucose homeostasis by hampering gluconeogenesis through phosphorylation and inhibition of a transcription factor FoxO1 [84,85]. mTORC2 indirectly acts as negative feedback of IRS through phosphorylating and stabilizing the ubiquitin ligase protein Fbw8, a member of the F-box protein family responsible for the degradation of inactive IRS1 in the cytosol, but the mechanism needs to be further elucidated [89].

The mTOR pathway plays a key role in the regulation of cellular growth and proliferation. The major cause for diet-induced obesity and insulin resistance is the overactivation of mTOR that down-regulates insulin signaling via negative feedback regulation through the phosphorylation of IRS1 at serine residues [81,86]. Thus, pharmacological intervention that can target the mTOR pathway to deactivate the negative feedback loop to reverse insulin resistance, maintain nutrient homeostasis, regulate cellular growth and proliferation will be useful in the prevention and/or treatment of diabetes. Future work should focus on enabling molecular insights related to mTOR function and regulation to develop clinically beneficial rational targeting of mTOR signaling.

### 3.7. AMP-Activated Protein Kinase

The AMP-activated protein kinase (AMPK) is a ubiquitously expressed serine/threonine kinase. AMPK is a central regulator of multiple metabolic pathways and is activated by diminished cellular energy state. AMPK has a heterotrimeric structure consisting of one catalytic subunit (α) and two regulatory subunits (β and ϒ). Each subunit of AMPK has different isoforms encoded by distinct genes, which are expressed specifically in tissue [90,91]. Activation of AMPK restores cellular energy homeostasis by invigorating catalytic processes to generate ATP while inhibiting anabolic processes [92]. Through phosphorylation of previous metabolic substrates and transcriptional regulators, AMPK plays a substantial role in glucose uptake by upregulating GLUT4 expression through phosphorylation of transcription repressor histone deacetylase (HDAC)5, mitochondrial biogenesis, and fatty acid oxidation while suppressing the synthesis of fatty acids, cholesterol, and protein [77,93,94].

AMPK is generally correlated with the increase of insulin sensitivity in the body and decrease of insulin resistance as it is the inhibitor of acute proinflammatory responses through reduced FAO. There is a close link between dysregulation of AMPK and insulin resistance in both rodents and humans [91]. AMPK mediates contraction provoked GLUT4 translocation and glucose uptake through an insulin-independent mechanism. The cellular energy sensor Sirtuin1 and AMPK regulate each other. AMPK precedes Sirtuin 1, and they are both components of adaptive responses to insulin resistance, oxidative stress, ER stresses, and inflammation [95] (Figure 1). AMPK can be activated by exercise and calorie restriction, and in contrast, it is downregulated in response to high glucose exposure. AGE induces glucose uptake impairment in skeletal muscle through a RAGE-mediated AMPK downregulation [96]. There is a positive synergism between AGE receptor 1 (AGER1), Sirtuin 1, and AMPK. Their combined effect is responsible for blocking AGE, sustaining the expression of each other, and providing a shield against the deleterious effects of redox imbalance. Thus, AMPK and Sirtuin 1 activation is associated with a wide array of beneficial effects for maintaining glucose homeostasis in an insulin-resistant state [59]. Multiple studies have demonstrated that insulin resistance and metabolic syndrome are associated with a decrease in AMPK activity, specifically in muscles and adipose tissues [97,98,99].

AMPK acts as a master metabolic regulator as it is required to restore energy balance, promote glucose uptake, and guard mitochondrial health [90,100]. Moreover, AMPK also inhibits proinflammatory responses and protects against obesity-induced insulin resistance [90,101]. These multifaceted physiological effects of AMPK make it an attractive target to ameliorate pathologies related to insulin resistance. Further insights into mechanisms of AMPK regulation, post-translational modifications, and the identification of additional AMPK substrates may lead to the development of more controlled pharmacological modulations.

### 3.8. AGE/RAGE/NF-κB Axis

Hyperglycemia is the characteristic feature of insulin resistance and one of the major factors contributing to diabetic complications, but apart from that, hyperglycemia is also involved in a series of chemical processes known as the Maillard reaction. In the Maillard reaction, reducing sugars nonenzymatically react with the amino group of proteins, lipids, the nucleic acid to produce AGE [102,103]. The complexity and diversity of AGE permanently modify the structure of proteins, plasma lipoproteins, cell membrane phospholipids, or DNA. AGE can induce the formation of aggregated proteins, which is the reason for the toxicity of AGE-modified polypeptides [104]. Mostly AGE production is endogenous, but primarily prooxidative dietary AGE can also supplement the risk for insulin resistance. AGE-mediated damage occurs through interaction with the multi-ligand, well-characterized cell surface pattern recognition receptor RAGE, which is ubiquitously expressed on several cell types [96]. RAGE is a significant mediator in the development of pancreatic β-cell dysfunction and the prognosis of diabetes. In the pancreas, AGE plays a mechanistic role in the aggregation of proteins to generate amyloid formation. Circulating AGE increases the expression of RAGE in the pancreas, which by engagement with toxic aggregates of different amyloidogenic derived proteins contributes to islet amyloidosis [8,37,105]. The key feature of islet amyloidosis is the pathological aggregation of IAPP, an endocrine hormone. The interaction of RAGE and IAPP activates the mediators of oxidative stress, inflammation, and apoptosis, which are the key features of IAPP induced β-cell cytotoxicity [37].

Chronic activation of RAGE by its ligands contributes to the upregulation and activation of NF-κB, a sequence-specific major transcription factor. NF-κB belongs to Rel family proteins and plays an important role in inflammation associated with insulin resistance [106]. On activation, NF-κB increases the expression of RAGE itself and proinflammatory cytokines, such as TNF-α, IL-1β, and IL-6, which trigger ER stress. Once triggered, the ER stress evokes the UPR to restore the cellular homeostasis, which increases intracellular ROS formation [107]. Inflammation, oxidative stress, and PKC activation lead to inhibition of IRS1 activity through its enhanced serine/threonine phosphorylation and reduced tyrosine phosphorylation, which results in impaired insulin signaling, as shown in Figure 1 [108,109]. NF-κB, specifically the NF-κB p65 subunit and NF-κB p105 subunit, bind to the promoter region of solute carrier family 2 member 4 (Slc2a4) gene, thus downregulating the transcription of GLUT4 expression [110,111].

The data herein show strong interdependency of hyperglycemia, insulin resistance and RAGE/ NF-κB mediated enhancement of inflammation and oxidative stress. There is a vicious cycle between inflammation and insulin resistance placing the AGE/RAGE/NF-κB axis at the nexus of this metabolic disorder. For future research development, the major challenge is the design of potential therapeutic approaches that mitigate NF-κB mediated pathological responses. This cannot be achieved without a complete understanding of NF-κB gene regulation, and the regulatory mechanisms involved in NF-κB activation to better understand the long-term effects of NF-κB modulating therapies.

## 4. Lipotoxicity, Inflammation, and Oxidative Stress

The correlation between lipotoxicity and insulin resistance is well established and involves multiple pathways [16]. Lipotoxicity is the consequence of excess calorie intake, fat accumulation, and increased lipolysis, which disrupts cellular homeostasis and causes metabolic pathway impairment in peripheral organs such as muscles, liver, pancreas, and adipose tissue [112]. The lipid spill-over of FFAs due to saturation of adipose tissue storage capacity leads to the accumulation of fatty acyl-coenzyme A, diacylglyceride (DAG), and ceramide in muscles [113]. Furthermore, prior studies have shown that these deleterious lipid intermediates have the greatest impact on the development of skeletal muscle insulin resistance, which is responsible for 70–80% of whole-body insulin-stimulated glucose uptake [114]. Among them, ceramide is a key lipotoxic player implicated as an antagonist of insulin action [115]. It is comprised of a sphingoid base coupled to a variable fatty acid side chain. The 4,5-trans-double bond in its sphingoid backbone bestows the unique biophysical properties that initiate the cellular stress responses, pro-apoptotic functions, cell growth arrest, and acts as a major inflammatory response messenger involved in the installation of muscle insulin resistance [116,117]. This enhanced level of ceramide interferes with muscle insulin sensitivity primarily through inhibition of the Akt/PKB activity [113,118]. Various studies reveal the link of high plasma ceramide levels with obese insulin-resistant states [119,120]. Both in vitro and in vivo studies evaluating the intracellular concentrations of ceramides showed a positive association between ceramide in lipotoxic situations and development of insulin resistance [116,121,122]. Studies evaluating tissue levels of ceramides by using liver [123], adipose tissues [124], and skeletal muscle [125] biopsies also reveal that ceramide level, hyperglycemia, and insulin resistance are positively correlated. There is a strong overlap between metabolic overload and inflammatory signaling pathways. The group of cytokines secreted by adipose tissues is known as adipokines [126]. These adipokines act as proinflammatory cytokines (such as IL-1, IL-6, and TNFα) that induce inflammation in the target tissue, which causes chemotactic invasion and infiltration of immune cells and further procreates the problem. The prolonged abnormal secretion of cytokines will ultimately disturb the delicate balance between inflammation and metabolic pathways [9,127]. One of the hallmarks of prolonged lipotoxicity is chronic low-grade metabolic inflammation, termed “metainflammation” [128]. The recognition of saturated fatty acids by the Toll-Like Receptor (TLR) family of pattern recognition receptors, specifically TLR4, is another mechanism associated with obesity induced metainflammation [129,130]. TLR4, expressed in insulin target tissues, plays a crucial role in the development of insulin resistance and inflammation. TLR4 triggers metabolic inflammation and insulin resistance during obesity by upregulating the transcription of proinflammatory genes and activating proinflammatory kinases JNK, IKK, and p38. These kinases, through inhibitory phosphorylation of insulin receptor substrate (IRS) on serine residues, impair insulin signal transduction [131]. A previous study revealed that TLR4 lies upstream of ceramide biosynthesis, which links lipid-induced inflammatory pathways to the antagonism of insulin action [132].

The activation of JNK and IKK, which induces insulin resistance through PKC, is the consequence of activated inflammatory signaling networks [127]. There are convincing data to support that JNK has a central role in inducing insulin resistance and type 2 diabetes [133]. JNK1 knockout mice exposed to a high-fat diet confers long-term metabolic protection from diet-induced obesity and display markedly improved insulin sensitivity with a normal life span [134]. Direct involvement of JNK in insulin signaling is through the JNK phosphorylation site on IRS1 at Ser**^307^**, which inhibits the interaction of IRS1 with the insulin receptor and constrains its tyrosine phosphorylation [133]. Furthermore, the interaction of PKC, JNK, and IKK will disrupt the metabolic homeostasis through the activation of kinases involved in serine phosphorylation of IRS and will inhibit insulin action [127]. These kinases will also activate the NF-κB signaling, which will result in a burst of inflammatory responses [127,135,136].

These immune responses are also linked to another key factor crucial for the integration of insulin resistance, which is known as ER stress. ER stress is accompanied by the accumulation of misfolded protein, which activates unfolded protein response (UPR) in the ER lumen [137]. The prolonged incapability to re-establish ER homeostasis results in the UPR-dependent activation of inflammation and eventually, apoptosis. Mitochondria also become hyperactive under the nutrition overload and produce more of their natural by-product, the reactive oxygen species. The redox imbalance occurs when the production of reactive oxygen molecules exceeds the capacity for their clearance [18,138]. Oxidative stress is the outcome of the overgeneration of ROS in mitochondria that induce ER stress. The oxidative stress generated by ROS production, in turn, produces more oxidative stress, thus resulting in a vicious cycle of spiraling oxidative stress condition, which results in ROS induced cellular components damage and trigger transcriptional changes that promote insulin resistance [139].

The excess production of reactive oxygen species directly stimulates the IKK/NF-κB, JNK pathways and damages the infrastructure of the cell through mitochondria-induced stress responses [140]. The impact of mitochondrial perturbations will lead to downregulation of the key regulator of mitochondrial biogenesis peroxisome proliferator-activated receptor-gamma coactivator 1α (PGC-1α) and will increase mitochondrial apoptosis susceptibility [16,141]. The chronic elevation of FFAs is linked to inflammation through activation of protein kinase C, IKK/NF-κB, JNK pathways, ER stress, redox imbalance and results in dysregulation in intracellular signaling, which ultimately leads to the pathological situation of insulin resistance [10,127]. The data presented herein provide directionality for the events involved in lipotoxicity, inflammation, and oxidative stress leading to insulin resistance. During the insulin-resistant state, there are a plethora of interconnected events taking place at a time. Therefore, it is necessary to target several pathways at the same time for the management of this condition. The maintenance of cellular homeostasis, mitochondrial quality, redox homeostasis, improving the inflammatory condition, and depletion of TLR4 protein expression might be attractive pharmacological targets to increase insulin sensitivity. Our understanding of mechanisms involved in insulin resistance suggests the use of antioxidants and pharmacological modulators suppress the chronic activation of pathways involved in the crossroad of lipotoxicity, inflammation and oxidative stress, leading to insulin resistance. Future studies delineating regulatory pathways controlling the onset of insulin resistance will aid in identifying new target genes and facilitate the design of novel therapeutic approaches. The understanding of mechanistic interactions between various kinases remains incomplete and needs to be evaluated further.

## 5. Concluding Remarks

Insulin resistance and hyperglycemia are the key pathophysiological processes of type 2 diabetes mellitus and diabetic complications [11]. The principal mechanism of insulin resistance is hyperglycemia-induced hyperproduction of AGE. The high circulatory levels of AGE lead to the ubiquitous expression of RAGE in insulin-sensitive tissues and pancreatic β-cells. β-cell dysfunction and insulin resistance are interdependent. Diminished pancreatic β-cells mass and dysfunction lead to a decline in insulin secretion and insulin insensitivity. Pancreatic β-cells toxicity is associated with the binding of toxic aggregates of AGE and IAPP to the multiligand receptor RAGE. As a consequence, the production of inflammatory cytokines, chemokines, oxidative stress, and activation of signaling cascades related to cellular toxicity results in beta cell damage and degradation [37,51,102]. The enhanced insulin demand to overcome the insulin-resistant state of the body from the pancreas with receding β-cell count further aggravates the condition.

AGE interaction with RAGE is also associated with peripheral insulin resistance with the induction of signaling cascades causing activation of the JNK pathway, IKKα/β, and master transcription factor NF-κB [104]. All these events are associated with chronic low-grade inflammation, ER stress inducing UPR, excess oxidative stress, activation of stress kinases leading to IRS serine phosphorylation, degradation, and downregulation of major glucose transporter GLUT4. The downregulation of AGER1 encoded by the dolichyl-diphosphooligosaccharide protein glycosyltransferase (DDOST) gene responsible for AGE degradation and AMPK repression responsible for GLUT4 expression through activation of transcription factor MEF2, are also critical in intensifying the insulin resistance state [96,105].

The afore-mentioned interconnected metabolic abnormalities underlying insulin-resistance mechanism leads to the pathological condition of diabetes mellitus. Multiple pathophysiological mechanisms underlying insulin resistance should be targeted and treated simultaneously to combat this metabolic dysfunction. This review concludes that future strategic interventions focused on reducing AGE production and downregulating RAGE expression along with targeting insulin sensitivity will be beneficial to counteract the insulin signaling pathway perturbations. This approach will be beneficial not only from the perspective of improved insulin sensitivity but also from the perspective of preserving the β-cell mass and function.
